# Silencing the ASI gustatory neuron pair increases expression of the stress-resistance gene *sod-3* in a *daf-16* and *daf-3* independent manner

**DOI:** 10.17912/W2W37V

**Published:** 2018-05-30

**Authors:** Peter Chisnell, Cynthia Kenyon

**Affiliations:** 1 Department of Biochemistry & Biophysics, University of California, San Francisco, San Francisco, CA 94143-2200, USA

**Figure 1.**
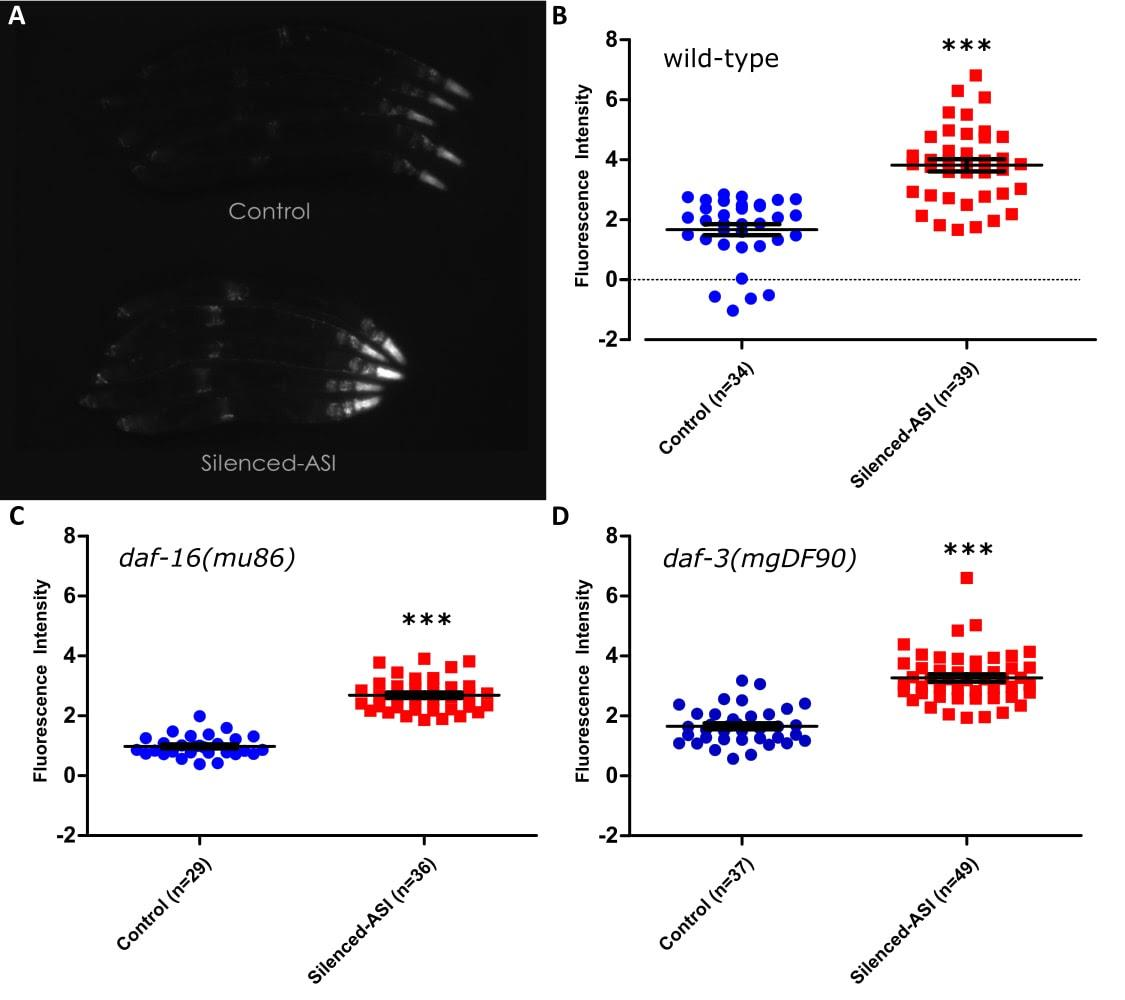


## Description

Ablation of the gustatory neuron pair ASI has been shown to extend lifespan, and this lifespan extension requires the function of the transcription factor *daf-16*/FOXO (Alcedo and Kenyon 2004). Given this relationship, we tested whether silencing the neuron pair ASI with the tetanus toxin light chain (Tetx), as opposed to ablating it, could increase the expression of a well- known DAF-16 target, the stress-resistance gene *sod-3* (Furuyama et al. 2000; Honda and Honda 1999). Tetanus toxin disrupts neurotransmission by blocking the release of both small clear-core vesicles and large dense-core vesicles, but should not affect communication via gap junctions (Schiavo et al. 1992; McMahon et al. 1992) . In order to measure the effects on *sod-3* expression, we measured GFP fluorescence in animals which expressed a p*sod-3*::GFP construct with or without the addition of GFP::Tetx expressed under the ASI-specific promoter p*gpa-4* (see methods; Libina et al. 2003). Animals with silenced ASI showed increased fluorescence in the wild- type background (Figure Panel A, B), as well as in loss of function mutants for the genes *daf-16* (Figure Panel C) or *daf-3* (Figure Panel D), all with P values <0.0001. This finding was unexpected for the following reasons: both genes are required for the lifespan extension caused by loss of signaling from DAF-7/TGFβ (Shaw et al. 2007), which is only secreted from ASI under normal laboratory conditions (Meisel et al. 2014), *daf-16* is required for the ablation of ASI to extend lifespan (Alcedo and Kenyon 2004), and optogenetic activation of ASI has been shown to decrease the activity of DAF-16 (Artan et al. 2016).

## Methods

Animals collected from the same plate were divided into experimental or control conditions based on expression or lack thereof of RFP. Animals were then paralyzed with 10mM levamisole for 10 minutes, corralled together into rows, and then imaged at 50x under bright field and to measure GFP fluorescence. Images contained ~5 animals per image. Light level and exposure time (108ms) were selected so as to provide the brightest pictures without any saturation and were kept constant both within and across experiments. To analyze the images in as unbiased a manner as possible, the entire body of each animal was outlined under a brightfield image in imageJ and then the average intensity of that outline was measured in the corresponding fluorescent image. A large section of the background fluorescence was measured and this number was subtracted from all measurements. Significance was measured by comparing the control and experimental group utilizing a two-tailed t-test. While the tetanus toxin construct itself was bound to GFP, the intensity of that construct was exceedingly dim and we required the use of an RFP co-injection marker to separate Tetx-expressing animals from controls at magnifications of 50x and lower. In addition, the increase in expression was not restricted to the ASI neuron pair alone but also appeared throughout the head as well as the nerve cord, parts of the vulva, and the tail, all locations the p*sod-3*::GFP construct is expressed under control conditions (Figure Panel A; Libina et al. 2003). Due to both these pieces of evidence, we conclude that the increased GFP measured is due to the effects of tetanus toxin in ASI.

## Reagents

CF4126: *muEx641*[pPC30(p*gpa-4*::GFP::Tetx) + p*unc-122*::RFP] CF4138: *muIs84* [pAD76(p*sod-3*::GFP)]; *muEx641* [pPC30(p*gpa-4*::GFP::Tetx) + p*unc-122*::RFP] CF4170: *daf-16*(*mu86*)I; *muIs84*[pAD76(p*sod-3*::gfp)]; *muEx641*[pPC30(p*gpa-4*::GFP::Tetx) + p*unc-122*::RFP] CF4172: *daf-3*(*mgDf90*)X; *muIs84*[pAD76(p*sod-3*::gfp)]; *muEx641*[pPC30(p*gpa-4*::GFP::Tetx) +p*unc-122*::RFP]
